# Perceived Utility and Characterization of Personal Google Search Histories to Detect Data Patterns Proximal to a Suicide Attempt in Individuals Who Previously Attempted Suicide: Pilot Cohort Study

**DOI:** 10.2196/27918

**Published:** 2021-05-06

**Authors:** Patricia A Areán, Abhishek Pratap, Honor Hsin, Tierney K Huppert, Karin E Hendricks, Patrick J Heagerty, Trevor Cohen, Courtney Bagge, Katherine Anne Comtois

**Affiliations:** 1 Department of Psychiatry and Behavioral Sciences University of Washington Seattle, WA United States; 2 ALACRITY Center University of Washington Seattle, WA United States; 3 Department of Biomedical Informatics and Medical Education University of Washington Seattle, WA United States; 4 Sage Bionetworks Seattle, WA United States; 5 Kaiser Permanente Northern California, CA United States; 6 Center for Suicide Prevention and Research University of Washington Seattle, WA United States; 7 University of South Alabama Mobile, AL United States; 8 School of Public Health University of Washington Seattle, WA United States; 9 Department of Psychiatry University of Michigan Medical Center Ann Arbor, MI United States; 10 VA Center for Clinical Management Research VA Ann Arbor Healthcare System Ann Arbor, MI United States

**Keywords:** real-world data, web searches, suicide risk factors, suicide detection, suicide, eHealth, internet, website, search history, risk, EHR, social media, behavior, mental health, personalized, online seeking behavior

## Abstract

**Background:**

Despite decades of research to better understand suicide risk and to develop detection and prevention methods, suicide is still one of the leading causes of death globally. While large-scale studies using real-world evidence from electronic health records can identify who is at risk, they have not been successful at pinpointing when someone is at risk. Personalized social media and online search history data, by contrast, could provide an ongoing real-world datastream revealing internal thoughts and personal states of mind.

**Objective:**

We conducted this study to determine the feasibility and acceptability of using personalized online information-seeking behavior in the identification of risk for suicide attempts.

**Methods:**

This was a cohort survey study to assess attitudes of participants with a prior suicide attempt about using web search data for suicide prevention purposes, dates of lifetime suicide attempts, and an optional one-time download of their past web searches on Google. The study was conducted at the University of Washington School of Medicine Psychiatry Research Offices. The main outcomes were participants’ opinions on internet search data for suicide prediction and intervention and any potential change in online information-seeking behavior proximal to a suicide attempt. Individualized nonparametric association analysis was used to assess the magnitude of difference in web search data features derived from time periods proximal (7, 15, 30, and 60 days) to the suicide attempts versus the typical (baseline) search behavior of participants.

**Results:**

A total of 62 participants who had attempted suicide in the past agreed to participate in the study. Internet search activity varied from person to person (median 2-24 searches per day). Changes in online search behavior proximal to suicide attempts were evident up to 60 days before attempt. For a subset of attempts (7/30, 23%) search features showed associations from 2 months to a week before the attempt. The top 3 search constructs associated with attempts were online searching patterns (9/30 attempts, 30%), semantic relatedness of search queries to suicide methods (7/30 attempts, 23%), and anger (7/30 attempts, 23%). Participants (40/59, 68%) indicated that use of this personalized web search data for prevention purposes was acceptable with noninvasive potential interventions such as connection to a real person (eg, friend, family member, or counselor); however, concerns were raised about detection accuracy, privacy, and the potential for overly invasive intervention.

**Conclusions:**

Changes in online search behavior may be a useful and acceptable means of detecting suicide risk. Personalized analysis of online information-seeking behavior showed notable changes in search behavior and search terms that are tied to early warning signs of suicide and are evident 2 months to 7 days before a suicide attempt.

## Introduction

Worldwide, suicide is the 18th leading cause of death, resulting in nearly 800,000 lives lost annually [1]. Suicide is the 10th leading cause of death in the US, with nearly 48,000 Americans dying by suicide in 2017 and 1,400,000 suicide attempts in the same year, costing the US approximately 69 billion dollars in 2015 [2,3]. Despite the high societal and personal costs of suicide and decades of research into suicide prevention, we still cannot accurately identify who may be at risk for death by suicide or when the risk is likely to be greatest [4,5].

One of the most important challenges facing suicide prevention researchers—as well as clinical providers—is to identify warning signs for suicidal behavior [6,7], which indicate when a specific individual is at heightened suicide risk in the near term (ie, within minutes, hours, or days) [6,8]. While warning signs developed by expert consensus [6] have been widely disseminated [9-11], research on the timeliness and utility of proposed warning signs has been sparse, and some proposed warning signs have recently been found to not predict imminent suicidal behavior [12]. This is partly because warning signs such as short-term increase in alcohol use [13,14], acute negative interpersonal life events [15], intensity of affective [8], cognitive responses, suicide-related communications, and prepping one’s personal affairs [12] may not be static variables (ie, they fluctuate over minutes, hours, or days) making traditional clinical risk assessment at a single health care contact imprecise [6]. Additionally, only one-third of mental health providers use suicide screens routinely [16]. An added complication is that, currently, known methods rely upon having a vulnerable individual self-disclose to and interact with systems (eg, health care, school) or individuals (eg, family, friends) in order to identify warning signs of suicidal behavior. Many people at high risk for suicide do not seek professional help because of lack of time, preference for other sources of help, or fear regarding how they will be treated in the health care system (eg, stigma and the potential trauma associated with in-patient hospitalization) [17-22]. For these reasons, methods that can identify proximal risk factors for suicide that do not depend on health care disclosure and can capture the fluctuating and individualized nature of suicide risk are needed [23].

Machine learning and natural language processing methods have recently been applied to social media data as a means of identifying suicide risk [24,25]. Social media provide a continuous stream of information about individuals’ daily lives that may be useful in capturing the dynamic nature of suicide risk, and some studies have found these data can be used to infer a person’s mental health as well as ongoing risk for suicide [23,26-29]. A disadvantage to using social media is that the age range of users is still very young [30,31]. Because of recent mishandling of social media data, people are also staying away from public social media platforms [32]. By contrast, 77% of the US population [33] seek information online through internet search engines. The information gleaned here is less likely to be biased, for example, by the desire to project a positive persona on social media [34], with search content focused on information gathering about personal concerns [35,36]. The use of internet search data may be an effective, private, immediate method of proximal suicide risk detection for individuals regardless of their contact with systems of care or self-reported disclosures to other persons.

The use of individualized web searches for proximal risk assessment of suicide attempts is also aligned with the Fluid Vulnerability Theory [29,37-39]. Fluid Vulnerability Theory proposes that suicide risk is a function of ever-changing interactions among multiple risk and protective factors, thus “an individual’s vulnerability to suicide is variable but nonetheless identifiable and quantifiable [37].” Some risk and protective factors are static or relatively stable (eg, gender, race, genetics, trauma, dispositional optimism) whereas others are state-based and dynamic (eg, mood, life stressors, insomnia, social support). While static risk factors are known to identify who is at risk, using personalized web searches as a source of real-time real-world behavior may potentially help uncover complex interplay of real-world risk factors associated with when someone is at greatest suicide risk.

However, one limitation to using search data for suicide risk prediction is the concern about privacy and the use of data not intended for public consumption. The intent of social media is to share life events and information publicly whereas search queries are often meant for personal, nonpublic use, and thus privacy concerns about using these data are important to understand [40]. Before embarking on a scaled exploration of these data for risk detection purposes, it is crucial to gain insight from people with lived experience of suicide about their comfort with their search data being used for suicide detection and prevention.

The purpose of this study is to examine the feasibility of using data from internet searches to identify suicide risk. The first objective of this study was to determine whether internet search behavior (frequency of search queries and queries categorized to known warning signs) were evident within 60 days of a documented suicide attempt. The second objective of this study was to determine how comfortable individuals with lived experience of suicide are with the use of internet search data for early identification.

## Methods

### Data Collection

#### Recruitment and Eligibility

Participants with a prior confirmed suicide attempt were recruited from an ongoing randomized clinical trial [41]. Inclusion criteria were (1) inpatient or emergency service admission, (2) lifetime suicide attempt and current hospital admission for suicidality or current hospital admission for a suicide attempt, and (3) consenting to study procedures. This study was approved by the institutional review board at the University of Washington. Data collection occurred from November 2017 to October 2019, and data analysis was conducted from October 2019 to August 2020.

#### Procedures

Participants were asked to complete a 30-minute semistructured interview about their concerns and suggestions for using internet search data as a means of preventing suicide. They were offered the option to provide a one-time confidential data download of their online Google search history. Participants who opted to participate in the study were reimbursed US $30 regardless of whether or not they agreed to share their Google search history.

#### Internet Search Data Collection

Google Takeout is a web-based interface developed by Google that allows users of Google apps to download their data into an exportable file. Using prior work [42], we created a web app, called gTAP [43], to allow participants to download their data without sharing personal Google account credentials. Only past Google search history was collected (Multimedia Appendix 1).

The Suicide Attempt and Self-Injury Count, a brief version of the Suicide Attempt Self-Injury Interview [44], has been widely used to determine suicide attempts in clinical trials [45-49] and was used to identify dates of suicide events and categorize events into suicide attempts and nonsuicidal acts.

#### Participant Survey

The interview was developed by the research team and focused on the acceptability of using internet information to detect the risk of suicide and to prevent suicide (Multimedia Appendix 2). Participants were asked to respond to the following question: “Technology companies use algorithms to predict who is at risk for suicide. Were you aware of this? Do you have any concerns or fears about how this information is collected, stored, and shared? How would you feel if they used your personal search data and/or what you have posted on social media to take action to prevent you and others in a similar situation from suicide? What do you see as the pros and cons?”

### Statistical Analysis

#### Search Data Featurization

Participants’ web searches were used to generate behavioral (online information seeking pattern) and semantic (meaning of search content) features. For behavioral features, we generated daily summary of participants' search history such as the average number of searches per day and the time of day when searches were conducted. For semantic features, we applied distributional models of semantics [50] to derive vector representations of participants’ queries, such that queries and words relating to similar concepts would be proximal in the resulting vector space. To do so, we used semantic vectors [51-53] and publicly available pretrained word embeddings [54] (Multimedia Appendix 3 and Multimedia Appendix 4). In order to map the user search queries to 9 empirically supported warning signs [12] and suicide-method preparation (which has not been previously examined using the present approach), we developed a set of cue terms. An iterative process of cue term definition, expert review of proximal queries, and refinement of the cue term set was used to generate the final set. We calculated a proximity score between the participants' search query (web search) and vector subspace derived from the cue terms representing a warning sign, using the Gram-Schmidt orthonormalization [55] procedure to ensure mutual orthogonality. The proximity score was estimated as the length of the projection of the query into this subspace, following the quantum disjunction method [56]. We used a conservative *z* score threshold ≥3.5 (corresponding *P*=.0002) to indicate a meaningful construct-to-query association relative to all search queries for each participant. The use of a threshold of this nature is required on account of the statistical properties of high-dimensional space—all vectors in this space have measurable similarity, but only proximal neighbors in the space indicate meaningful similarity [55,57,58], which should be well above what would be anticipated by chance on account of the high probability of randomly instantiated vectors being mutually close-to-orthogonal [59,60]. Furthermore, this threshold was supported empirically by inspection of the relationships between queries that fell above and the themes of interest in a subset of the data (Multimedia Appendix 4).

#### Association Analysis

Because of a small sample size and high level of heterogeneity in web search data across participants, we used an individualized analytical approach (Multimedia Appendix 4) to assess the magnitude of difference between web search data proximal (7, 15, 30, and 60 days) to the suicide attempts versus the typical (baseline) search behavior of participants. In order to show the difference between the observed search feature score prior to the time of a suicide attempt and a typical baseline of the search feature for an individual, we computed 2 versions of a standardized value for the search score at the time of the event. First, we presented the *z* score defined as the search score at the observed event time (based on a chosen time window), standardized using the mean and variance associated with a patient-specific reference distribution characterizing the full range of observed search scores for an individual. This highlighted how far the observed score at the event time was from typical scores. Second, given that the reference search score distributions may be highly skewed or multimodal, we also computed the empirical reference percentile for each search feature for the event time (suicide attempt). Specifically, we used an empirical reference distribution per search feature to compute the percent of typical individual-specific search feature values that fell either above or below the corresponding search feature value for an observed attempt. A percentile calculation is a form of nonparametric standardization and is termed a *placement value* for classification problems [61]. To show either extremely low or high values, we calculated the symmetrized placement value defined as the minimum of the upper or lower tail probability for the observed attempt score. The participant-specific reference distributions were generated using a nonparametric Monte Carlo simulation [62]. All statistical analysis was performed using the R [63] statistical programming language.

#### Survey Data Analysis

Participants’ responses to structured survey questions were summarized using summary statistics. Semistructured responses were transcribed verbatim during participant interviews and anonymized prior to analysis. We used a mixed methods approach, combining quantitative and qualitative data with the function of expansion [64], which allowed inductive qualitative data to provide the *why* to questions uncovered by the quantitative data. Qualitative data were analyzed from a constructivist perspective using thematic analysis after all interviews were completed [65]. Two coders (a psychologist and a psychiatrist) independently extracted initial themes from survey responses. Themes were reviewed by a third coder, a clinical research assistant who had performed all participant interviews. Data were iteratively reviewed (open coded) and collapsed to mutually exclusive themes (axial coding) until saturation was achieved (ie, when no new themes emerged) [66]. Triangulation [67] of quantitative and qualitative data allowed for convergence of themes and a more comprehensive understanding of willingness to use search data for prevention. Illustrative quotes and themes are provided for a qualitative data audit trail. This study was conducted in accordance with SRQR (Standards for Reporting Qualitative Research) [68].

## Results

### Sample Characteristics

**Figure 1:**
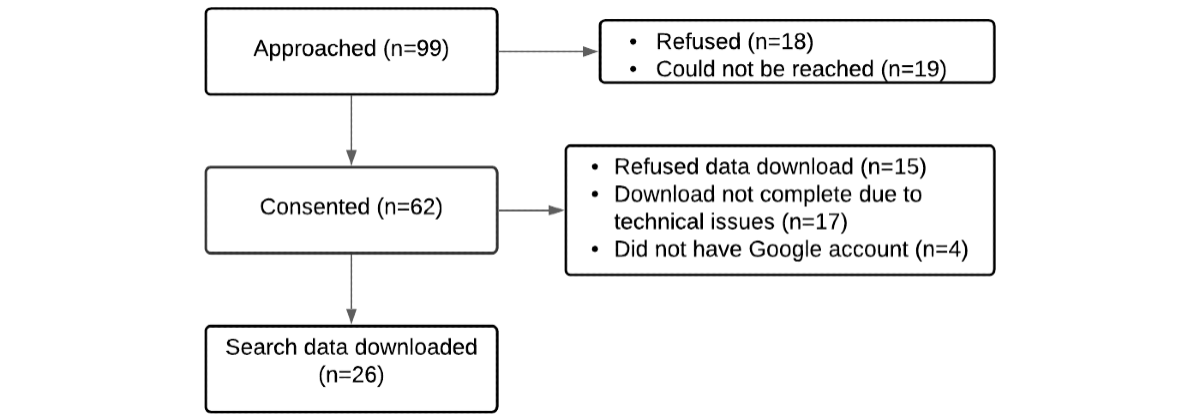
Study CONSORT flow diagram.

Of 150 individuals, 99 were eligible to participate in this study and were approached, and 62 consented to participate in the qualitative interview. Of the 62 who consented, 26 (42%) were able to provide web search data. Reasons for not providing data were technical issues in downloading search data (17/62, 27%), unwillingness to share Google searches (15/62, 24%), and not having a Google account (4/62, 6%) (Figure 1). The cohort that consented to participate in the qualitative interview consisted of predominantly White individuals (43/62, 69%), and 53.2% were male (33/62). The mean age of the cohort was 34.9 years with 79% (49/62) having at least some college education. No significant differences in demographics were observed between the participants who completed qualitative interviews (n=62) and the final subset (n=26) from whom the Google search data was obtained for age (*P*=.18), gender (*P*=.58), race (*P*=.83), marital status (*P*=.94), education (*P*=.96), and income (*P*=.98) (Table 1). A total of 71 lifetime suicide attempts were reported by the full sample (n=62). The precision of the estimated attempt date varied from the exact attempt date (33/71, 46%) to within 2 weeks (11/71, 15%). To align the suicide attempt period to a proximal web search window, we only used the attempt dates that were rated as accurate within 2 weeks (n=44). Of these, retrospective web search data were available for 30 attempts.

**Table 1:** Demographic characteristics.

Characteristics		Approached (n=99)	Consented (n=62)	Search data downloaded (n=26)	*P* value
Age (years) at enrollment, mean (SD)		33.10 (12.45)	34.94 (13.15)	29.62 (9.15)	.18
**Gender, n (%)**					.58
	Male	50 (50.5)	33 (53.2)	15 (57.7)	
	Female	38 (38.4)	21 (33.9)	5 (19.2)	
	Other	5 (5.1)	4 (6.5)	3 (11.5)	
	Transgender	6 (6.1)	4 (6.5)	3 (11.5)	
**Race, n (%)**					.83
	White	66 (66.7)	43 (69.4)	21 (80.8)	
	Mixed	20 (20.2)	14 (22.6)	3 (11.5)	
	Asian	7 (7.1)	2 (3.2)	2 (7.7)	
	Black or African American	4 (4.0)	3 (4.8)	0 (0.0)	
	American Indian or Alaska Native	1 (1.0)	0 (0.0)	0 (0.0)	
	Native Hawaiian or Other Pacific Islander	1 (1.0)	0 (0.0)	0 (0.0)	
**Marital status, n (%)**					.94
	Single/never married	72 (72.7)	42 (67.7)	18 (69.2)	
	Divorced	12 (12.1)	10 (16.1)	3 (11.5)	
	Married	9 (9.1)	4 (6.5)	2 (7.7)	
	Separated	5 (5.1)	5 (8.1)	3 (11.5)	
	Widowed	1 (1.0)	1 (1.6)	0 (0.0)	
**Education, n (%)**					.96
	Some college, associate’s degree, or technical training	53 (53.5)	33 (53.2)	16 (61.5)	
	Bachelor’s or graduate degree	22 (22.2)	16 (25.8)	4 (15.4)	
	High school graduate or GED	15 (15.2)	9 (14.5)	5 (19.2)	
	Some high school	7 (7.1)	3 (4.8)	1 (3.8)	
	Other	2 (2.0)	1 (1.6)	0 (0.0)	
**Income^a^, n (%)**					.98
	Less than $5000	9 (10.8)	5 (9.4)	3 (14.3)	
	$5000-9999	11 (13.3)	8 (15.1)	2 (9.5)	
	$10,000-24,999	23 (27.7)	12 (22.6)	4 (19.0)	
	$25,000-49,999	21 (25.3)	16 (30.2)	7 (33.3)	
	More than $50,000	15 (18.1)	9 (17.0)	4 (19.0)	
	None	4 (4.8)	3 (5.7)	1 (4.8)	

^a^Data were missing for n=16, n=9, and n=5 individuals for approached, consented, and data downloaded, respectively.

### Search Data

#### Summary

**Figure 2:**
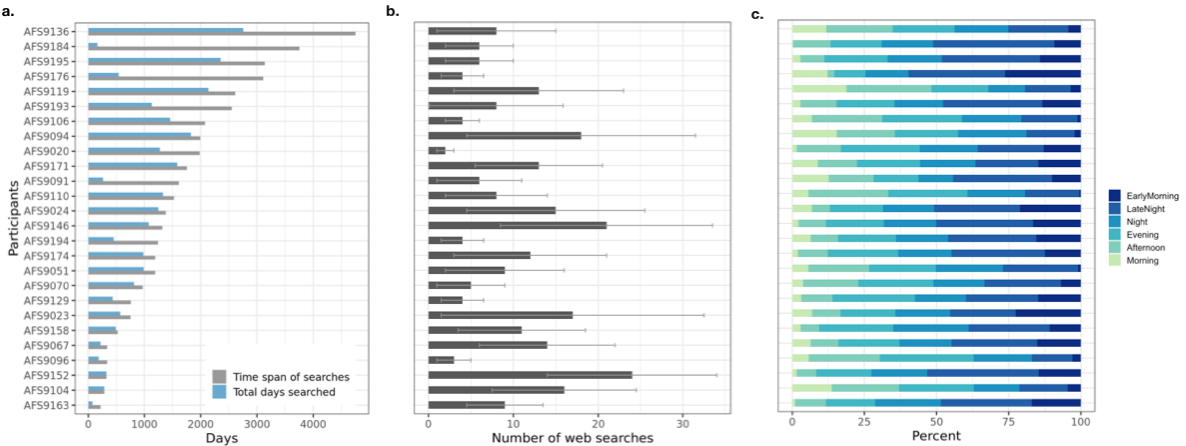
Search data characteristics across participants: (a) span (in days) of search data collected from participants (in grey) and the number of days (blue) on which participants made at least one search. (b) Median daily number of web searches performed by the participants. The error bars indicate the 25th and 75th percentile. (c) Proportions of participants' web searches stratified by time of day.

In total, search history data for 24,397 days were collected with 349,922 individual search queries from 26 study participants. The median time span of the search history data across participants was 1348 days (range 220-4752 days); however, the actual number of days when participants searched online was much lower than the data collection period and varied widely (median 898.5 days, range 75-2759 days) (Figure 2a). Analysis generated 11 high-level search constructs related to participants’ online information-seeking behavior (ie, search behavior features) and search content proximity scores for 10 suicide warning signs (ie, semantic features) (Multimedia Appendix 3).

#### Search Behavior Features

Our analysis revealed idiosyncratic online information seeking behavior across individuals; the number of searches per day varied between 2 searches and 24 searches (median 8.5 searches). The time of day when participants conducted online searches also varied (morning: 0%-4.35%; late night: 0%-37.5%; Figure 2b,c).

#### Semantic Features

Of the semantic search content that we mapped to known suicide warning signs, we identified a small proportion of search queries (median 1.2%, range 0.06%-21.47%) with a proximity *z* score ≥3.5 that mapped onto 1 or more of the 10 warning signs. At times, queries meeting this threshold were observed in close proximity to a suicide attempt (within days to weeks). Table 2 provides a representative selection of highly ranked queries (based on *z* scores) for each warning sign, revealing content suggestive of premeditation, preparation, substance use, stressors, underlying mental state, and help-seeking behavior.

**Table 2:** Cue term sets developed to represent selected warning signs and a subset of top search queries that map to each of the warning signs.

Warning sign	Cue terms	Retrieved search queries
Alcohol use	whiskey; alcohol; aa; wine; alcoholic; beer	“aa meetings”; “how much beer to get drunk”; “wine hangover vs. hard alcohol”; “alcohol poisoning”; “alcoholics anonymous”^a^
Preparation of personal affairs	will; affairs; suicide+note	“writing a suicide note”; “living will”; “write your will online”
Suicide communication	hotline; help; suicide+communicate	“what does suicide hotline do”; “suicide crisis text line”; “suicide text line”; “emergency room si suicidal ideation”
Suicide methods (preparation)	overdose; gun; lethal	“sleeping pill overdose suicide”; “is ambien lethal”; “where can I get suicide pills”^a^; “where to buy a gun in Seattle”; “cheap guns”^a^
Burdensomeness	burden	“discussing work burdens marriage”^a^
No reason to live	hopeless; live; persist	“I don’t want to live anymore”
Anger	hostile; rage; anger	“fits of rage”; “depression and rage”; “serious anger marijuana”
Anxiety	scared; fearful; afraid; anxiety; anxious; jittery;	“ocd anxiety”; “apprehensive”^a^; “social anxiety”; “marijuana for anxiety”; “why do I have so much anxiety”; “phobia of diseases”^a^
Emptiness	numb; hollow; feeling+empty	“I feel so empty”; “I like the feeling of being sad”
Interpersonal problem	conflict; divorce; fight; breakup; loss	“final divorce decree cost”; “infidelity and custody”^a^; “how much child support if spouse loses job”^a^; “divorce”

^a^Found using distributional semantic approaches (ie, queries do not contain any of the manually defined cue terms) illustrating the capacity of distributional semantics approaches to identify related concepts expressed in different terms.

### Association Between Search Data Features and Suicide Attempts

**Figure 3:**
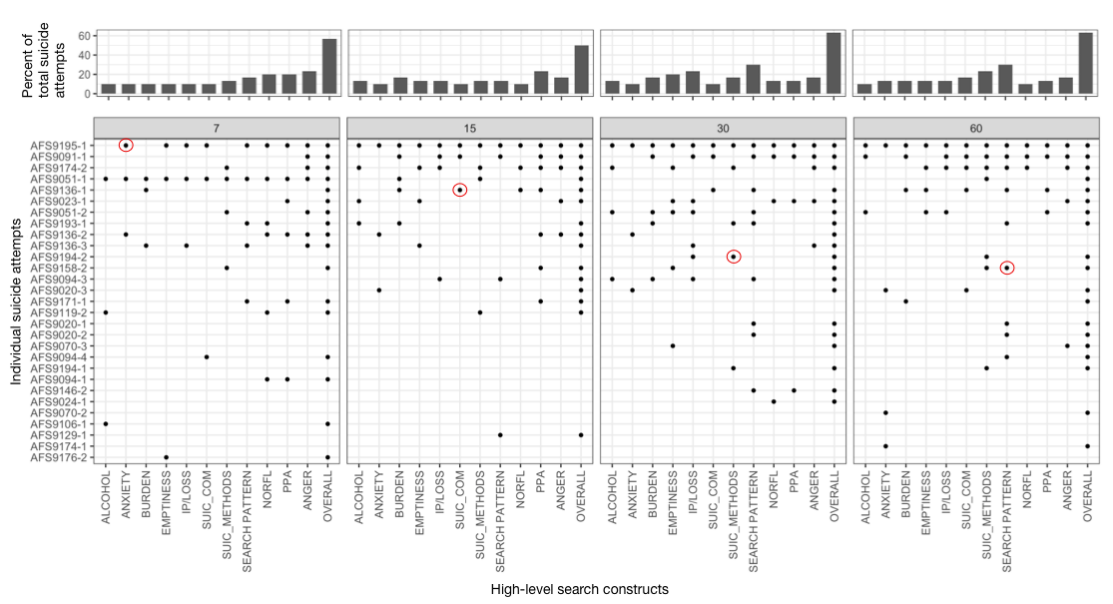
Summary of individualized association analysis for 11 high-level search constructs over 4 suicide attempt–proximal periods: (a) 7, (b) 15 (c) 30, and (d) 60 days.

**Figure 4:**
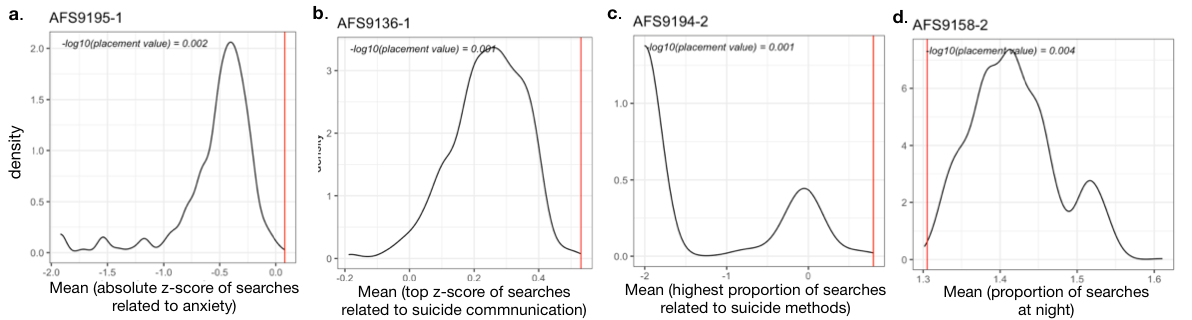
Baseline distributions for 4 example search features (each indicated by a red circle in Figure 3): (a) anxiety – 7-day proximal period; (b) suicide communication – 15-day proximal period; (c) suicide methods – 30-day proximal period; and (d) searches at night – 60-day proximal period. The red line indicates the value of the search feature in the corresponding time period proximal to a suicide attempt.

On average, 58% of attempts (n=30; range 15/30, 50% to 19/30, 63%) were found to be associated (–log_10_(placement value)≥2) with at least one search feature in 1 of the 4 proximal time periods (7, 15, 30, and 60 days). Figure 3 shows the summary of individualized association analysis highlighting the specific search constructs proximally associated with suicide attempts. Notably, for 23% attempts (7/30), a prolonged association with proximal search features (from 60 days) was observed, indicating an extended period of potentially high-risk online search behavior. For the majority of attempts, we observed a high degree of variation in search constructs and the proximal time period in which they were associated with attempts. The constructs associated the most attempts (across time windows) were online search patterns (9/30 attempts, 30%), semantic similarity of search queries to suicide methods (7/30, 23%), and anger (7/30, 23%). Figure 4 shows features associated with 4 individual suicide attempts (one per proximal window) where the search behaviors of the participants were found to be markedly different (–log_10_(placement value)≥2) from their typical (ie, baseline) behavior.

### Perceived Acceptability and Feasibility of Internet Search–Based Prediction of Suicide Risk

Three primary themes were identified regarding the acceptability of using search history for suicide prevention: utility, accuracy, and privacy (Table 3): 68% (40/59) thought using internet search history for suicide detection and prevention could be useful; many participant (34/59, 58%) raised concerns about the accuracy of detection, in particular concerns of false positives and their potential consequences; and 32% of participants (19/59) were concerned about the intrusion of privacy.

**Table 3:** Illustrative quotes of participant responses to use of internet history in suicide prevention.

Theme	Illustrative quotation	Respondents reporting the theme, n (%)
Useful	“It’d be a good way to help people get resources that they don’t otherwise know about.” “I think it’s good. I think people would be more open online than how they are in one-to-one in-person situations.” “I think it sounds great. They’re already using algorithms to optimize search, [so] might as well do it for something good.” “Potentially it could be enough to 'break the cycle' of suicidal thoughts.”	40/59 (68)
Detection accuracy concerns	“No problem with that as long as they did it right. I wouldn’t want the SWAT team to show up at my door...” “Companies don't understand the context of the situation, try to do the right thing, but...it makes people want to shut people out because they overreact.” “I don’t think I would mind because I know what I’m getting myself into. They should work on the algorithm before implementing it into the general population though.” “I like how they use trigger/key words to dump all these resources on you, but I think they need to improve it.”	34/59 (58)
Privacy concerns	“I'm chronically in private mode, because I don't want Google or tech other companies knowing I'm looking at this. If I'm ever in public, I don't want my search results to be seen by others.” “Feels like I am being spied on.” “I would be a little upset about that. Seems like an invasion of privacy.” “[As] we become more transparent on the internet, search history or data could be used [with] malicious intentions, planting bombs for the future.”	19/59 (32)

When presented with potential prevention interventions, participants favored interventions that provided a direct link to either a crisis counselor (35/61, 57%), friends or family (33/60, 55%), peers (30/61, 49%), or to a self-guided meditation video (33/61, 54%) (Multimedia Appendix 5). Interventions that simply provided a hotline number, with suggestions to reach out, or an inspirational video were not as favorable. When asked if they had seen interventions such as Google’s links to a suicide prevention hotline, only 53% (33/62) said they saw the link, and of those, only one person acted on it. Participants were allowed to voluntarily opt out of answering any questions in the qualitative survey, and if they did so for a specific question, they were not counted in the denominator of that question's response percentages. Although 62 participants consented to the qualitative interview and provided some form of response in the interview, up to 3 participants opted out of specific questions at various times for declining to answer or feeling too distressed to continue the interview.

## Discussion

### General

This is one of the first studies to examine and describe the nature of individualized internet search data with an eye toward suicide prevention. We found that while search queries and behavior do change prior to suicide attempts, there is considerable variation between individuals, with some participants searching online more frequently, and others seeking information online sporadically prior to attempts. Additionally, search queries over time are highly individualized, and for some attempts, changes in search behavior and queries related to risk are evident 60 days before the attempt, with a majority evident 2 weeks before the attempt. Search content associated with risk windows also varied, although some content was highly prevalent across time points such as queries expressing anger or suicide methods. Although these findings suggest that the use of internet searches for risk prediction will be complicated due to the intraindividual variation, it may still be possible to develop a personalized temporal risk profile or a digital phenotype [69] linked to suicide-related behaviors. Previous research has found that personalized models lead to more accurate prediction of clinical states [70]. In the present study, individualized risk assessment analysis identified as much as 63% of attempts (19/30) based on changes in search behavior and queries.

We found that participants felt using internet search data to predict and intervene in suicide was potentially helpful, but they also harbored some important reservations. Participants felt that any intervention based on search history or social media algorithms would need to be highly accurate and respect personal privacy. The interventions themselves should be active (link to a friend), rather than passive (suggestion to contact a hotline). Importantly, participants were particularly concerned about the use of emergency services as a means of intervention.

This study represents the first step in understanding the potential utility of online search data for suicide prevention. The next steps will require a study with a much larger sample size due to the intraindividual variation in search signal differences, in addition to interindividual variation in search terms and search behaviors prior to attempts. Expansion of the semantic feature space may also further refine predictive signals. While these results demonstrate that a personalized analytical approach can identify patterns of search behaviors that are evident up to 2 months before an attempt, larger studies are needed to assess potential representational bias and further refine high-risk signatures from online search data.

### Limitations

This study is a preliminary cohort study and thus has limitations. First, this was a small sample. Although individualized analysis of web search data indicates the potential benefits for understanding real-world risk factors of suicide and when someone may be at a higher risk, future research should explore the cohort-level predictive ability of data, including optimization of analytical parameters (eg, selection of threshold to indicate a meaningful association between web searches and suicide risk factors). Second, participants in this study were at very high suicide risk with both a lifetime suicide attempt and a recent episode requiring hospitalization. A larger prospective study of people with varying levels of suicide risk, including those without a history of suicidal ideation, is warranted to ensure that the search patterns and terms found here are unique to the imminent risk of suicide. Understanding the perspectives of other individuals on the sharing of web search data and the appropriateness of intervention will also be crucial prior to any deployment of prediction algorithms and related suicide prevention efforts. Finally, we asked participants their perspectives about hypothetical intervention scenarios based on internet search informed risk prediction. It is likely that participants’ acceptability of such interventions will differ when they are faced with them during a crisis event.

### Conclusion

Although this is a preliminary study, the findings are promising and suggest a potentially useful and timely method for utilizing search data for detecting the risk of suicide. If handled appropriately, this method of risk detection is seen by those with a lived experience of suicide as an acceptable method of detection. Suicide is a serious public health problem, one that has the potential to escalate during these times of health, societal and economic challenges. Methods that can quickly identify and intervene to prevent a suicide event could help prevent and reduce the public health burden of suicide.

## Appendix

Multimedia Appendix 1 A brief schematic overview of the gTAP workflow.

Multimedia Appendix 2 Interview Guide for Exploring Technology for Suicide Prevention AFS Sub-Study.

Multimedia Appendix 3 Description of Search Data Features.

Multimedia Appendix 4 Schematic overview of search data featurization and non-parametric association analysis to compare the difference between a typical “baseline” search behavior to a proximal period before the documented suicide attempt.

Multimedia Appendix 5 Survey participants were asked to rate helpfulness and comfortableness with each of the following potential intervention options. Values represent percentages of all participants that responded to each question.
