# Supplementary material for: Perceived Utility and Characterization of Personal Google Search Histories to Detect Data Patterns Proximal to a Suicide Attempt in Individuals Who Previously Attempted Suicide: Pilot Cohort Study
Source: J Med Internet Res. 2021 May 6;23(5):e27918. doi: 10.2196/27918 (PMC8138707; doi:10.2196/27918)

Multimedia Appendix 1: A brief schematic overview of the gTAP workflow - participant initiated GTO data download, acquisition, sensitive data redaction, and secure storage of data for downstream analysis by study researchers. Once a participant initiates GTO data download through the gTAP interface, Google prepares and delivers the data to the participant's Google Drive using the Google Takeout feature that is available to any Google account holder. The gTAP can then download the data from their Google Drive using the prior approved OAuth encrypted credentials. The credentials allowing investigators access to data were stored for short-term (max 24 hours). Data are stored on participants' Google drive only to allow the app to download the data. Before the data was stored for any analysis, it was de-identified to the extent possible using a data loss prevention (DLP) API to redact any individual's sensitive search queries such as SSN, driver's license number, and other identifiable information.

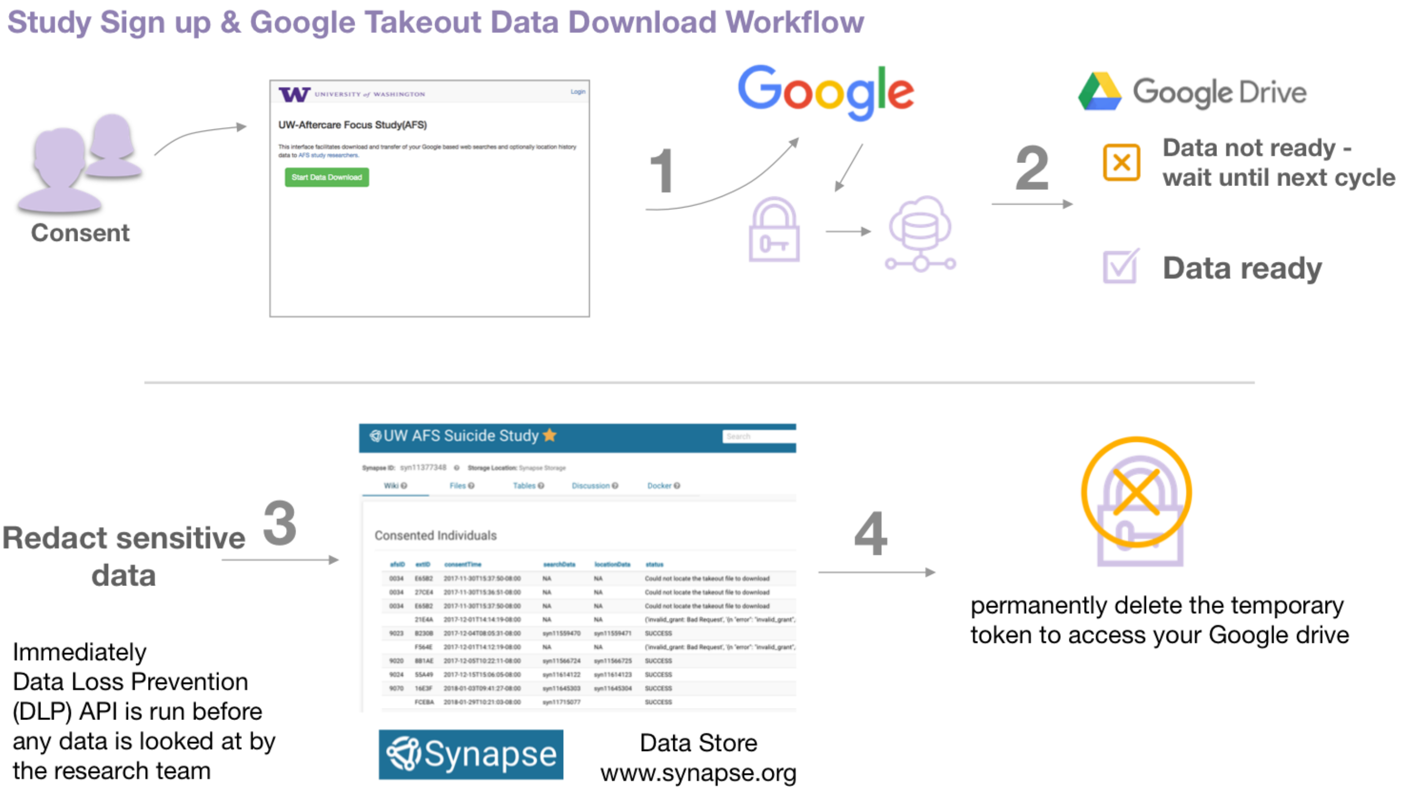

Supplement: Multimedia Appendix 1 [file jmir_v23i5e27918_app1.pdf]
