# Supplementary material for: Perceived Utility and Characterization of Personal Google Search Histories to Detect Data Patterns Proximal to a Suicide Attempt in Individuals Who Previously Attempted Suicide: Pilot Cohort Study
Source: J Med Internet Res. 2021 May 6;23(5):e27918. doi: 10.2196/27918 (PMC8138707; doi:10.2196/27918)
Supplement: Multimedia Appendix 2 [file jmir_v23i5e27918_app2.pdf]

Multimedia Appendix 2: Interview Guide for Exploring Technology for Suicide Prevention AFS Sub-Study

Thank you so much for agreeing to talk to us about how you search the web, your use of social media platforms such as Facebook, Twitter, etc. and your thoughts on general information technology.

We are interested in learning more about how people search the web and use social media when they are in distress and during the time period when they may begin to actively think of ending their lives.

We would like see if we can identify whether people are at risk of suicide from how they use the internet and develop interventions to help people in times of distress.

For the first part of this sub-study, we will ask you questions about how you use web searches and social media (such as Google or Facebook), as well as what your thoughts are about the utility of these data to find solutions for proactively helping people who are suffering in times of distress.

As with the other interviews you have completed with the Aftercare Focus Study, what you tell me today will be kept confidential and you are free to skip any questions you do not want to answer.

Do you use the following search tools or social media platforms? If so, which ones and how frequently? Primarily on your phone, computer, or both? Primarily for work, personal use, or both?

		Never	Monthly or yearly	Weekly	Daily	Several times per day	Phone=1 Computer=2 Both=3	Work=1 Personal=2 Both=3
	**Search tools**							
1.	Google	0	1	2	3	4	(1a)	(1b)
2.	Bing	0	1	2	3	4	(2a)	(2b)
3.	Yahoo	0	1	2	3	4	(3a)	(3b)
4.	Other(4aa):	0	1	2	3	4	(4a)	(4b)
5.	Other(5aa):	0	1	2	3	4	(5a)	(5b)
	**Social media**							
6.	Facebook	0	1	2	3	4	(6a)	(6b)
		**Never**	**Monthly or yearly**	**Weekly**	**Daily**	**Several times per day**	**Phone=1 Computer=2 Both =3**	**Work=1 Personal=2 Both=3**
7.	Instagram	0	1	2	3	4	(7a)	(7b)
8.	Pinterest	0	1	2	3	4	(8a)	(8b)
9.	Twitter	0	1	2	3	4	(9a)	(9b)

10. Reddit	0	1	2	3	4	(10a)	(10b)
11. Snapchat	0	1	2	3	4	(11a)	(11b)
12. LinkedIn	0	1	2	3	4	(12a)	(12b)
12.1 YouTube	0	1	2	3	4	(12.1a)	(12.1b)
12.2 Tumblr	0	1	2	3	4	(12.2a)	(12.2b)
13. Other(13aa):	0	1	2	3	4	(13a)	(13b)
14. Other(14aa):	0	1	2	3	4	(14a)	(14b)

How often do you use Amazon? Primarily on your phone, computer, or both? Primarily for work, personal use, or both?

	**Never**	**Monthly or yearly**	**Weekly**	**Daily**	**Several times per day**	**Phone=1 Computer=2 Both =3**	**Work=1 Personal=2 Both=3**
15. Amazon	0	1	2	3	4	(15a)	(15b)

(16) Have you ever noticed that before? What is your opinion about this?

Google, Facebook, Amazon, Apple (Siri) and other sites use your search data in a lot of ways. Based on your Google search history, Google tries to optimize how to create the best search of webpages for you, to maximize the chance that you will get what you are looking for from your first search attempt. Likewise, all these companies use this information to send you advertisements that are most likely to appeal to you.

(17) Do your opinions about this vary depending on the website?
INTERVIEWER: If necessary, prompt for whether they feel differently about certain companies (e.g., Google vs. Twitter) or type of website (e.g., search vs. social media using their search history).

(18) When you were searching for something (either in an internet browser or in social media), have you ever seen a message box at the top of the results page with the suicide prevention lifeline phone number? (18.1) ______(1=Yes, 0=No)

(18.2) (If yes) Do you remember what you were searching for?

(18.3) (If yes) Did this message box ever prompt you to call the lifeline or act differently than what you originally intended?

(19.1) Technology companies remove and censor content, including banning or removing posts or images and “sensitivity screening” (blurring images until a user explicitly indicates they want to view the

image). Were you aware of this? What do you think about technology companies doing this to prevent suicide or self-harm? What do you see as the pros and cons?

(20) Technology companies use algorithms *(if unclear, prompt with: how Amazon or Netflix knows what to recommend to you or how Facebook knows who to recommend as a friend)* to predict who is at risk for suicide. Were you aware of this? Do you have any concerns or fears about how this information is collected, stored and shared? How would you feel if they used your personal search data and/or what you have posted on social media to take action to prevent you and others in a similar situation from suicide? What do you see as the pros and cons?

Imagine if you were feeling very low and you were searching the internet or looking at social media. I am going to list some options that could be offered to you based on your search history or social media activity. For each option, we would like to know how helpful and how comfortable you would be with each option if a search engine or social media site offered these through your search results or social media feed. We would also like to know your thoughts or opinions about each option.

INTERVIEWER: Use the following rating scales and ask for additional thoughts or opinions for each option.

Helpful

5=Very helpful, 4= Somewhat helpful, 3=Neutral, 2=Somewhat unhelpful, 1=Very unhelpful

Comfortable

5=Very comfortable, 4=Somewhat comfortable, 3=Neutral, 2=Uncomfortable, 1=Very uncomfortable

(21) …a prompt for you to call the crisis line phone number [appeared in your search results or social media feed]

(21a)Helpful ______ (21b)Comfortable______

Helpful

5=Very helpful, 4= Somewhat helpful, 3=Neutral, 2=Somewhat unhelpful, 1=Very unhelpful

Comfortable

5=Very comfortable, 4=Somewhat comfortable, 3=Neutral, 2=Uncomfortable, 1=Very uncomfortable

(22) …a prompt for you to call a friend or family member's phone number [appeared in your search results or social media feed]

(22a)Helpful ______ (22b)Comfortable ______

(23) …an inspirational video offering a message of support [appeared in your search results or social media feed]

(23a)Helpful ______ (23b) Comfortable ______

(24) …an instructional video for guided meditation or deep breathing exercises [appeared in your search results or social media feed]

(24a)Helpful ______ (24b) Comfortable ______

(25) …an option to connect via direct chat or videoconference with a crisis counselor, who could help you feel better [appeared in your search results or social media feed]

(25a)Helpful ______ (25b) Comfortable ______

(26) …an option to connect via direct chat or videoconference with a friend or family member, who could help you feel better [appeared in your search results or social media feed]

(26a)Helpful ______ (26b) Comfortable ______

Helpful
5=Very helpful, 4= Somewhat helpful, 3=Neutral, 2=Somewhat unhelpful, 1=Very unhelpful

Comfortable
5=Very comfortable, 4=Somewhat comfortable, 3=Neutral, 2=Uncomfortable, 1=Very uncomfortable

(27) …an option to connect via direct chat or videoconference with someone you don't know, such as a peer or someone else who has experienced suicidal ideation or other mental health problems, who could help you feel better [appeared in your search results or social media feed]

(27a)Helpful ______ (27b) Comfortable ______

(28) Do you share your [computer/phone] with anyone else? ______(1=Yes, 0=No)

(28.1) Computer: ______(1=Yes, 0=No)

(28.2) Phone: ______(1=Yes, 0=No)

(28a)If YES, with whom do you share your [computer/phone]? Does sharing a [computer/phone] with someone else change your opinion about the previous options? In other words, would you want a different type of help if you were sharing vs. not sharing your [computer/phone]?

(29) What, if anything, do you do differently when you post something you know others can view on Facebook or Twitter, versus using a search engine for searching the internet?

(30) Does your internet use get monitored – by your employer, housing manager, parent, or someone else? ______(1=Yes, 0=No) If YES, who?____

(30a)Does this matter to you? In other words, would you want different "help" options (similar to those I mentioned above) because your internet use is monitored?

Researchers (like us) are interested in better understanding your answers to the questions I've just asked you. To do so, we would access information about your past online activity. We are interested in how willing you would be to share this information if requested by a specific website and/or a research study focused on suicide prevention in general.
Remember, we will not access any of this information unless you have given us explicit permission to do so.

INTERVIEWER: Please note comments but you do not have to probe for them.

How likely would you be to share your web searches (i.e., what you typed into the search engine, not the results that you received) with a research study focused on preventing suicide?

**Search tools**	**N/A**	**Definitely not**	**Probably not**	**Maybe**	**Probably**	**Definitely**
32. In general	-8	0	1	2	3	4
33. Google	-8	0	1	2	3	4
34. Bing	-8	0	1	2	3	4
35. Yahoo	-8	0	1	2	3	4
36. Other(36aa):	-8	0	1	2	3	4
37. Other(37aa):	-8	0	1	2	3	4

(38)Comments:

How likely would you be to consent to sharing your GPS location data with a research study focused on preventing suicide?

	**Definitely not**	**Probably not**	**Maybe**	**Probably**	**Definitely**
39. Share GPS location data	0	1	2	3	4

(40)Comments:

How likely would you be to share your public and private profile, postings, story, wall, feed, etc. with a research study?
INTERVIEWER: For this question, include public posts plus what they choose to share on that specific site.

	Social media	N/A	Definitely not	Probably not	Maybe	Probably	Definitely
41.	In general	-8	0	1	2	3	4
42.	Facebook	-8	0	1	2	3	4
43.	Instagram	-8	0	1	2	3	4
44.	Pinterest	-8	0	1	2	3	4
45.	Twitter	-8	0	1	2	3	4
46.	Snapchat	-8	0	1	2	3	4
47.	Reddit	-8	0	1	2	3	4
48.	LinkedIn	-8	0	1	2	3	4
48.1	YouTube	-8	0	1	2	3	4
48.2	Tumblr	-8	0	1	2	3	4
49.	Other(49aa):	-8	0	1	2	3	4
50.	Other(50aa):	-8	0	1	2	3	4

(51)Comments:

(31) What suggestions do you have for how technology companies could best help you and others in the future to prevent suicide?

(52) Any other feedback about this interview, sharing information for research, web searches, GPS, or social media data?
