# Supplementary material for: Perceived Utility and Characterization of Personal Google Search Histories to Detect Data Patterns Proximal to a Suicide Attempt in Individuals Who Previously Attempted Suicide: Pilot Cohort Study
Source: J Med Internet Res. 2021 May 6;23(5):e27918. doi: 10.2196/27918 (PMC8138707; doi:10.2196/27918)
Supplement: Multimedia Appendix 3 [file jmir_v23i5e27918_app3.pdf]

Multimedia Appendix 3: Description of Search Data Features

	Feature Type	Daily Feature Aggregation	Aggregate statistics in the window period (7, 15, 30, 60 days)
	Search Behavior Features		
1	Days without searches	N.A	Sum
2	Total number of searches	Sum	Mean<br>Minimum Value<br>Maximum Value<br>Standard Deviation
	Prop of searches by the time of day		
3	Early morning (4-7.59 AM)	Percent	
4	Morning (8-11.59 AM)	Percent	
5	Afternoon (12-3.59 PM)	Percent	
6	Evening (4-7.59 PM)	Percent	
7	Night (8-11.59 PM)	Percent	
8	Late Night (12-3.59 AM)	Percent	
	Semantic Features<br>*(Derived by mapping search queries to 10 known warning signs of suicide)*		
9	Alcohol	*For each sematic feature*<br>Mean Z-score<br>Max Z-Score<br>Min Z-Score<br>Max absolute Z-Score	
10	Anger		
11	Anxiety		
12	Burden		
13	Emptiness		
14	Interpersonal Loss		

15	No Reason for Living	Min absolute Z-Score	
16	Preparation of Personal Affairs		
17	Suicide Communication		
18	Suicide Methods		
