# Supplementary material for: Perceived Utility and Characterization of Personal Google Search Histories to Detect Data Patterns Proximal to a Suicide Attempt in Individuals Who Previously Attempted Suicide: Pilot Cohort Study
Source: J Med Internet Res. 2021 May 6;23(5):e27918. doi: 10.2196/27918 (PMC8138707; doi:10.2196/27918)
Supplement: Multimedia Appendix 4 [file jmir_v23i5e27918_app4.pdf]

Multimedia Appendix 4: Schematic overview of search data featurization and non-parametric association analysis to compare the difference between a typical "baseline" search behavior to a proximal period before the documented suicide attempt.

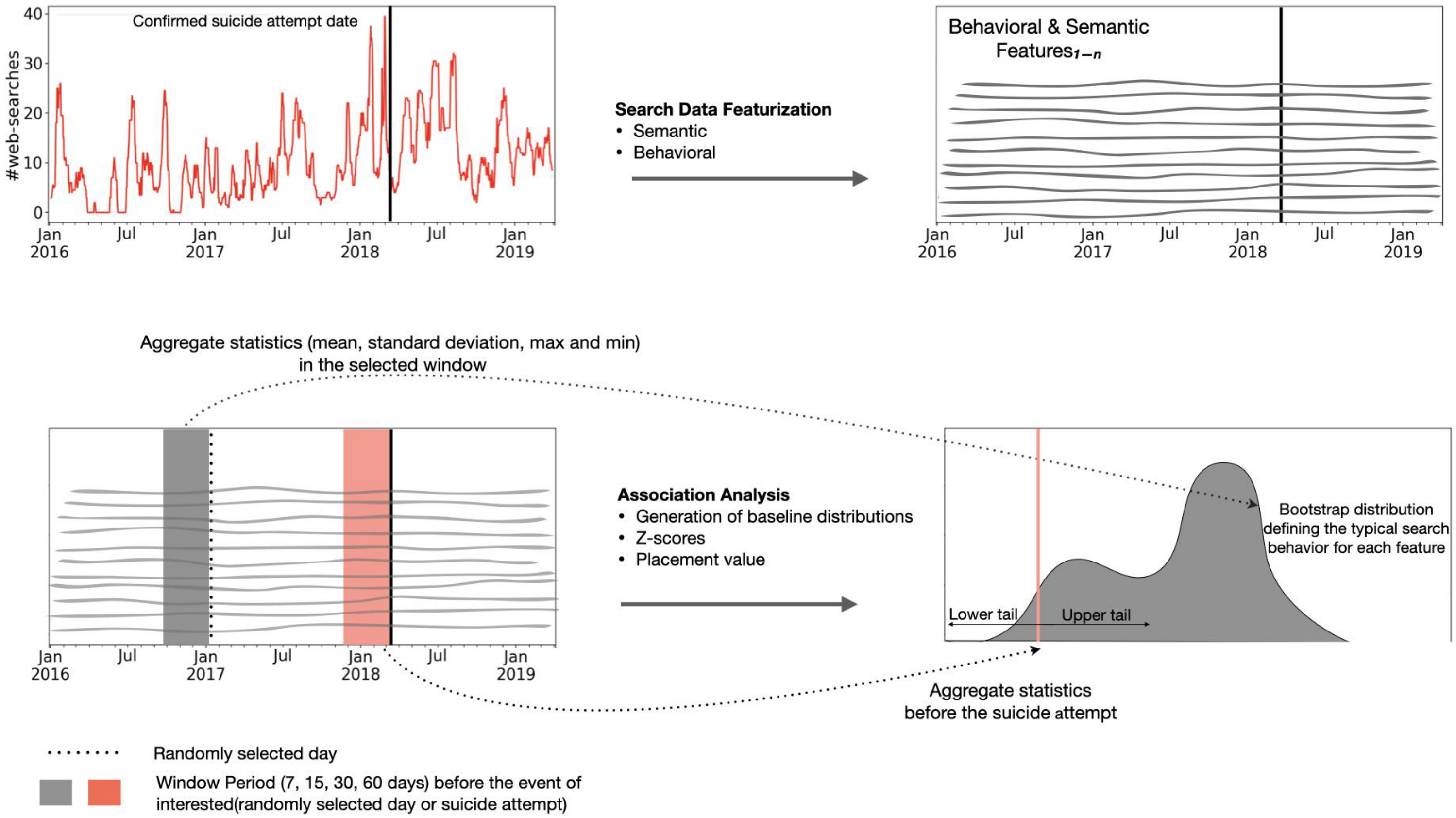

An open-source Semantic vectors package was used for generating semantic features[1]. Semantic features were estimated using the proximity score which estimates the semantic relatedness between a particular query and a warning sign on the basis of the distance between the vector representations of the cue terms representing a warning sign (see Table 2 in the manuscript) and the vector representation of a particular query. Using pre-trained word embeddings (vector representations of words derived from a neural network trained to predict words in proximity to an observed word), these query vectors were generated as the linear sum of the word embeddings for words they contain, Queries were represented as vectors by adding the pre-trained word vectors for words they contain, using Inverse Document Frequency weighting (to reduce the influence of frequently occurring terms which tend to be less semantically informative), and with subsequent normalization of the vectors to unit length. For each of the warning signs, a subspace (a region of interest of semantic vector space) was generated composed of a

set of mutually orthogonal vectors derived from the word embeddings for a set of cue terms using the Gram-Schmidt orthogonalization process. The proximity score for a query vector was then estimated as the length of its projection into the subspace representing a particular warning sign, following a procedure originally described by Widdows and Peters in their seminal work on quantum logic in semantic spaces [2].

In order to generate the participant-specific reference “baseline” distribution per search feature, we used a Monte-Carlo simulation-based approach[4]. A total of 1000 days were randomly sampled with replacement from an individual’s search history. Next, the aggregate statistics (mean, standard deviation, maximum, and minimum) were computed for all search features across each proximal time window (7, 15, 30, and 60 days) before the randomly selected day to generate baseline distributions for each participant. These individualized reference distributions were used to assess potential differences between search features proximal to a suicide attempt and typical search behavior. All statistical analysis was performed using the R[5] statistical programming language.

1. *Semantic vectors - https://github.com/semanticvectors/semanticvectors*
2. *Widdows, Dominic, and Stanley Peters. "Word vectors and quantum logic: Experiments with negation and disjunction." Mathematics of language 8.141-154 (2003).*
3. *Semantic Vectors: a Scalable Open Source Package and Online Technology Management Application Widdows D, Ferraro K LREC, 2008*
4. Raychaudhuri, S. Introduction to Monte Carlo simulation. in *2008 Winter Simulation Conference* 91–100 (IEEE, 2008).
5. R: The R Project for Statistical Computing. http://www.R-project.org/.
