# Supplementary material for: Perceived Utility and Characterization of Personal Google Search Histories to Detect Data Patterns Proximal to a Suicide Attempt in Individuals Who Previously Attempted Suicide: Pilot Cohort Study
Source: J Med Internet Res. 2021 May 6;23(5):e27918. doi: 10.2196/27918 (PMC8138707; doi:10.2196/27918)
Supplement: Multimedia Appendix 5 [file jmir_v23i5e27918_app5.pdf]

Multimedia Appendix 5: Survey participants were asked to rate helpfulness and comfortableness with each of the following potential intervention options. Values represent percentages of all participants that responded to each question.

How helpful and how comfortable would you be with each option if a search engine or social media site offered these through your search results or social media feed?

**A prompt for you to call the crisis line phone number:**					
	Very Helpful (%)	Somewhat Helpful (%)	Neutral (%)	Somewhat Unhelpful (%)	Very Unhelpful (%)
	9.8	31.1	24.6	18.0	16.4
	Very Comfortable (%)	Somewhat Comfortable (%)	Neutral (%)	Somewhat Uncomfortable (%)	Very Uncomfortable (%)
	27.9	19.7	24.6	16.4	11.5
**A prompt for you to call a friend or family member's phone number:**					
	Very Helpful (%)	Somewhat Helpful (%)	Neutral (%)	Somewhat Unhelpful (%)	Very Unhelpful (%)
	23.0	27.9	26.2	11.5	11.5
	Very Comfortable (%)	Somewhat Comfortable (%)	Neutral (%)	Somewhat Uncomfortable (%)	Very Uncomfortable (%)
	26.2	21.3	23	9.8	19.7
**An inspirational video offering a message of support:**					
	Very Helpful (%)	Somewhat Helpful (%)	Neutral (%)	Somewhat Unhelpful (%)	Very Unhelpful (%)
	15.0	21.7	28.3	18.3	16.7
	Very Comfortable (%)	Somewhat Comfortable (%)	Neutral (%)	Somewhat Uncomfortable (%)	Very Uncomfortable (%)
	38.3	21.7	16.7	13.3	10
**An instructional video for guided meditation or deep breathing exercises:**					
	Very Helpful (%)	Somewhat Helpful (%)	Neutral (%)	Somewhat Unhelpful (%)	Very Unhelpful (%)
	19.7	34.4	18.0	11.5	16.4
	Very Comfortable (%)	Somewhat Comfortable (%)	Neutral (%)	Somewhat Uncomfortable	Very Uncomfortable (%)

				(%)	
	32.8	29.5	26.2	4.9	6.6
**An option to connect via direct chat or videoconference with a crisis counselor, who could help you feel better:**					
	Very Helpful (%)	Somewhat Helpful (%)	Neutral (%)	Somewhat Unhelpful (%)	Very Unhelpful (%)
	29.5	27.9	16.4	14.8	11.5
	Very Comfortable (%)	Somewhat Comfortable (%)	Neutral (%)	Somewhat Uncomfortable (%)	Very Uncomfortable (%)
	19.7	23.0	21.3	21.3	14.8
**An option to connect via direct chat or videoconference with a friend or family member, who could help you feel better:**					
	Very Helpful (%)	Somewhat Helpful (%)	Neutral (%)	Somewhat Unhelpful (%)	Very Unhelpful (%)
	25.0	30.0	18.3	10.0	16.7
	Very Comfortable (%)	Somewhat Comfortable (%)	Neutral (%)	Somewhat Uncomfortable (%)	Very Uncomfortable (%)
	26.7	13.3	18.3	18.3	23.3
**An option to connect via direct chat or videoconference with someone you don't know, such as a peer or someone else who has experienced suicidal ideation or other mental health problems, who could help you feel better:**					
	Very Helpful (%)	Somewhat Helpful (%)	Neutral (%)	Somewhat Unhelpful (%)	Very Unhelpful (%)
	29.5	19.7	27.9	8.2	14.8
	Very Comfortable (%)	Somewhat Comfortable (%)	Neutral (%)	Somewhat Uncomfortable (%)	Very Uncomfortable (%)
	16.4	18.0	32.8	16.4	16.4
